# OLFM4, LCN2 and FAM3B as Potential Biomarkers for Gut–Brain Axis Dysfunction in IBD-MDD Comorbidity

**DOI:** 10.3390/genes17101245

**Published:** 2026-10-09

**Authors:** Fang Li, Junmei Zhao, Boyang Song, Jiheng Wang, Jing Zhang

**Affiliations:** 1 Translational Research on Cell and Gene Therapy for Higher Education Institutions for Shaanxi Key Laboratory of Higher Education Institutions, Xi’an Medical University, Xi’an 710021, China; 2 Medical College of Yan’an University, Yan’ an University, Yan’an 716000, China; 3 Department of Pharmacy, Xi’an Medical University, Xi’an 710021, China

**Keywords:** inflammatory bowel disease, major depressive disorder, biomarkers, gut–brain axis, machine learning, OLFM4, LCN2, FAM3B

## Abstract

Background: Inflammatory bowel disease (IBD) and major depressive disorder (MDD) exhibit bidirectional pathogenic interactions through the gut–immune–brain axis, but robust common biomarkers remain elusive. This study aimed to identify the core hub genes and their conserved signaling pathways in IBD-MDD comorbidity. Methods: We analyzed two transcriptome datasets, GSE117993 (pediatric IBD biopsy) and GSE98793 (MDD whole blood), to screen for shared differentially expressed genes (DEGs). Multi-algorithm machine learning, marker pathway correlation analysis, immune infiltration assessment and ROC validation were performed. Results: A total of 25 overlapping DEGs were identified, and three common core genes, OLFM4, LCN2, and FAM3B, were unanimously selected. Marker pathway analysis revealed that these genes were significantly associated with conserved inflammatory cascades in both diseases, including TNF/NF-κB and JAK-STAT signaling pathways, as well as interferon response and metabolic stress pathways. Analysis of immune infiltration indicated an association with neutrophils, M1 macrophages, and other immune subsets. The combined three-gene signature shows preliminary diagnostic performance within these datasets for IBD and MDD. Conclusions: This study identifies OLFM4/LCN2/FAM3B as potential shared biomarkers hinting at molecular links between IBD and MDD, providing new insights into candidate shared inflammatory mechanisms underlying gut–immune–brain axis dysfunction.

## 1. Introduction

Inflammatory bowel disease (IBD), including Crohn’s disease (CD) and ulcerative colitis (UC), is a chronic immune-mediated gastrointestinal disease with increasing prevalence worldwide, posing a significant public health burden [1]. Current therapeutic strategies centered on targeted biological agents have significantly improved mucosal healing. However, approximately 40% of patients have no response to treatment or gradually lose their response, and a large proportion of patients with clinical remission have refractory extraintestinal manifestations [2]. Major depressive disorder (MDD) is the most common and clinically influential neuropsychiatric comorbidity of IBD [3]. A growing body of clinical evidence supports a bidirectional pathogenic interaction between these two conditions rather than a passive psychological response to chronic disease [4,5]. IBD patients have a significantly increased lifetime risk of MDD, and pre-existing depressive symptoms in turn increase the susceptibility to the onset of IBD [6,7,8]. Comorbid MDD significantly aggravates IBD activity, increases the risk of disease attack, hospitalization and surgical intervention, and reduces treatment compliance, which seriously affects the long-term clinical outcome of IBD patients.

The gut–immune–brain axis is a key bidirectional signaling network connecting intestinal inflammation, peripheral immune dysregulation and central nervous system dysfunction, which is the basis of IBD-MDD comorbidity [9]. Chronic intestinal inflammation in IBD drives persistent systemic inflammation spillover, disrupting the integrity of the blood–brain barrier, and triggering neuroinflammatory responses that ultimately promote the occurrence of depression-like behavior and cognitive abnormalities [10]. Emerging genetic and bioinformatics studies have initially identified genetic pleiotropy and overlapping immune dysregulation pathways between IBD and MDD, while gut microbiota disorders and metabolic disorders further exacerbate the disease interaction [11,12]. However, existing studies mainly focus on macroscopic microbial alterations and single disease mechanism exploration. The candidate shared transcriptional signatures and key regulatory genes that may link intestinal inflammatory injury and neuropsychiatric dysfunction remain to be elucidated, and there is a lack of candidate molecular signatures to aid mechanistic interpretation of molecular crosstalk between IBD and MDD.

To fill these key research gaps, this study integrated two independent public transcriptome datasets, including GSE117993 (pediatric IBD endoscopic biopsy samples) and GSE98793 (whole blood samples from MDD patients), to screen for shared differentially expressed genes associated with IBD-MDD comorbidity. A variety of bioinformatics strategies including Metascape functional enrichment analysis, multi-algorithm machine learning feature selection, immune infiltration assessment and ROC diagnosis verification were used to screen and verify the core hub genes. Finally, we identified three potential shared gene signatures (OLFM4, LCN2, FAM3B) associated with molecular crosstalk between IBD and MDD. This study aims to explore candidate shared molecular clues for gut–immune–brain axis dysfunction, based on two independent disease datasets without true comorbid patients.

## 2. Methods

### 2.1. Data Acquisition

Two publicly available transcriptome datasets were retrieved from the Gene Expression Omnibus (GEO) database. The GSE117993 dataset contains raw count based RNA-seq profiles of endoscopic mucosal biopsy samples from pediatric inflammatory bowel disease (IBD) patients and non-IBD controls, including 135 IBD and 55 non-IBD samples. The GSE98793 dataset provides transcriptome expression data of whole blood in patients with major depressive disorder (MDD) and healthy individuals, including 128 MDD patients and 64 control samples. The original expression matrix was normalized and log2 transformed. No obvious outlier samples with abnormal expression distribution or incomplete expression data were identified, so all samples of the dataset were retained for subsequent analysis.

### 2.2. Identification of Differentially Expressed Genes

Differential expression analyses were performed independently for IBD and MDD cohorts using cohort-specific thresholds. For IBD RNA-seq count dataset (GSE117993), the DESeq2 package was used to screen differentially expressed genes (DEGs), and the cut-off criterion was set as <(|log_2_FC| > 1, *p* < 0.05). For MDD whole blood microarray dataset (GSE98793), transcriptional perturbations are generally subtle in peripheral blood; therefore, we set |log_2_FC| > 0.05 and nominal *p* < 0.05 to capture potentially meaningful disease-associated signals. Of note, nominal unadjusted *p* values were used for exploratory DEG screening, which may increase false-positive risk; subsequent multi-machine learning filtering and ROC analyses were applied to prioritize promising candidate genes. We subsequently performed Venn intersection analysis to screen for overlapping DEGs dysregulated in IBD and MDD and defined them as candidate genes for gut–brain axis mediated comorbidity.

### 2.3. Functional Enrichment and Cluster Analysis

Metascape (version v3.5.20250101, https://metascape.org, accessed on 16 April 2025) [13] was used to perform multi-database functional enrichment and cluster analysis, and the biological functions and pathway characteristics of shared IBD-MDD DEGs were systematically annotated. Multiple authoritative annotation resources such as Gene Ontology Biological Process (GO-BP), Reactome classical pathway, NABA matrix body tag, cell type tag, DisGeNET disease annotation and PaGenBase tissue-specific gene dataset were integrated. Functional terms with *p* < 0.05 were considered to have statistically significant enrichment. Enrichment results were visualized by bubble plots to show enrichment significance (−log10 *p*-value), annotated gene counts, and overall expression change trends based on z scores.

### 2.4. Multi-Machine Learning Screening of Hub Genes

In order to screen stable and general diagnostic signature genes from the 25 shared DEGs, we applied multiple supervised machine learning algorithms, including least absolute shrinkage and selection operator (LASSO) regression, random forest (RF) [14], eXtreme Gradient Boosting (XGB), gradient boosting machine (GBM), decision tree model (DT) and Support Vector Machine (SVM) [15]. Feature selection was performed separately in the IBD and MDD cohorts. LASSO regression and 10-fold cross-validation were used to screen the redundant genes with low contribution. The 1se criterion was used to determine the optimal gene combination. XGBoost and GBM algorithms quantify the feature importance of candidate genes based on model classification contributions. Stepwise regression and RMSE curve validation were further used to evaluate the stability of the feature screening results. The shuffled plot shows consistent genes screened by multiple algorithms in each cohort. Cross-cohort cross-analysis finally identified three core shared hub genes that were consistently identified in the IBD and MDD datasets.

### 2.5. Hallmark Pathway Correlation Analysis

Genome-wide Pearson correlation analysis was performed to explore the regulatory landscape of the three hub genes in IBD and MDD transcriptomic profiles. Correlation coefficients and corresponding *p*-values were calculated between each core gene and all detectable transcripts. Signature gene set enrichment and correlation cluster analysis further identified conserved signaling pathways regulated by OLFM4, LCN2 and FAM3B in gut inflammation and neuropsychiatric disorders. Overall pathway enrichment patterns and correlation distributions were visualized by integrating bubble-heat maps.

### 2.6. Immune Microenvironment Analysis

Based on standardized transcriptome data, CIBERSORT with the standard LM22 leukocyte signature matrix was applied separately to the IBD intestinal biopsy dataset and the MDD whole blood dataset to estimate relative fractions of 22 immune cell subsets. Pearson correlation analysis was performed to evaluate statistical associations between hub gene expression and estimated immune cell proportions; correlation coefficients, 95% confidence intervals and *p*-values were calculated and visualized in forest plots. For IBD intestinal samples we refer to intestinal immune microenvironment; for MDD whole-blood samples we describe peripheral immune cell composition.

### 2.7. Diagnostic Predictive Model Construction and Validation

Receiver operating characteristic (ROC) curve analysis was performed to evaluate the discriminatory efficacy of individual hub genes and the combined three-gene signature for IBD and MDD diagnosis. The area under the curve (AUC) was calculated to quantify the sensitivity and specificity of single-gene and multi-gene diagnostic models. The combined predictive model was constructed based on the three core hub genes to verify its universal diagnostic potential for distinguishing IBD and MDD from corresponding control samples.

## 3. Results

### 3.1. Screening of Differentially Expressed Genes Shared by IBD and MDD

**Figure 1:**
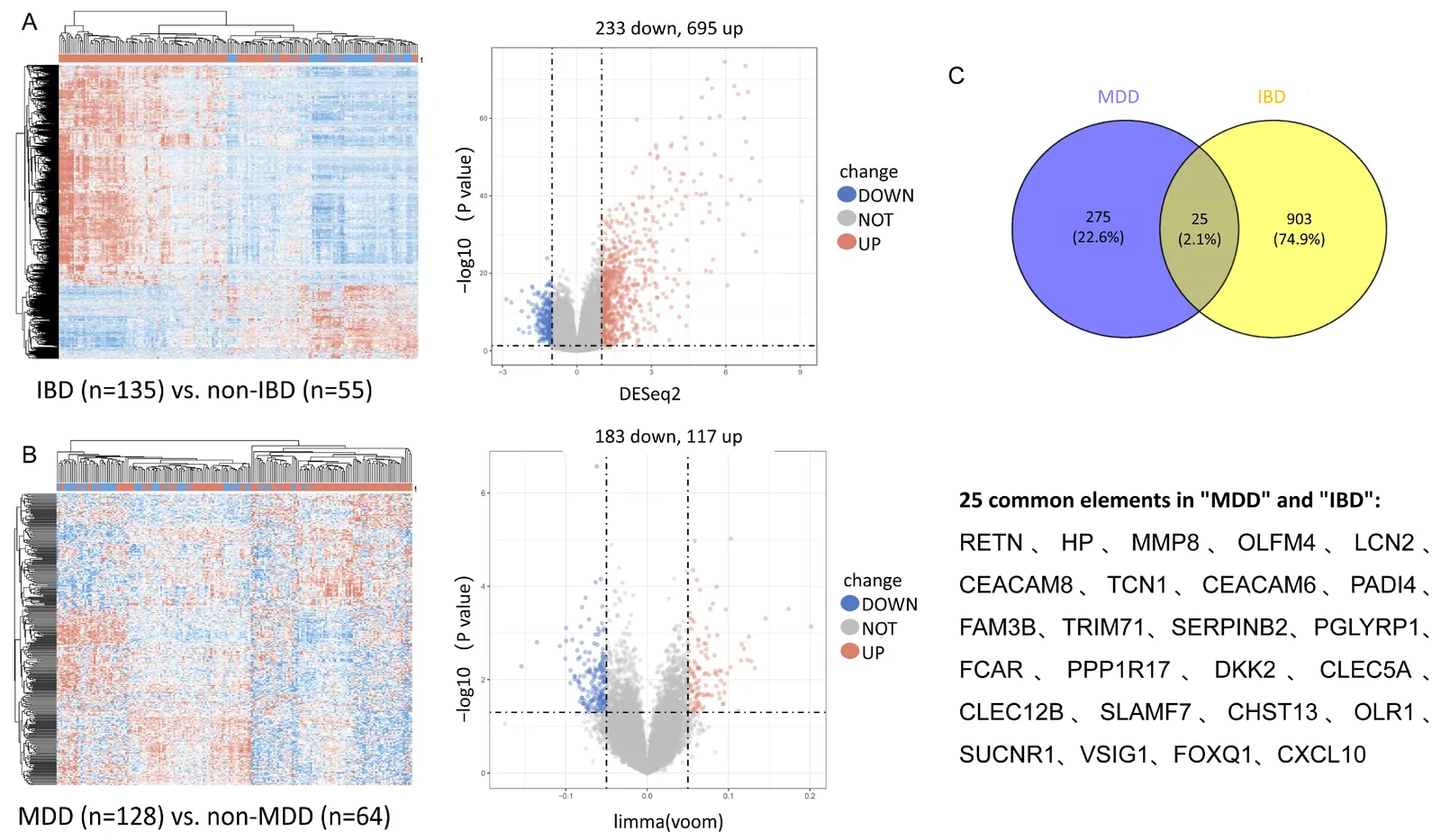
Screening of DEGs in IBD and MDD transcriptome datasets and acquisition of overlapping candidate genes for gut–brain axis research. (**A**) Expression heatmap and volcano plot of DEGs for IBD (*n* = 135) vs non-IBD (*n* = 55). DESeq2 identified 695 upregulated and 233 downregulated DEGs. (**B**) Expression heatmap and volcano plot of DEGs for MDD (*n* = 128) vs non-MDD (*n* = 64). Limma-voom algorithm obtained 117 upregulated and 183 downregulated DEGs. (**C**) Venn diagram showing 25 overlapping dysregulated DEGs between IBD-related DEGs and MDD-related DEGs; full gene list of 25 shared genes was listed on the right panel.

To identify common molecular changes in IBD and MDD comorbidities through the gut–brain axis, we performed differential expression analyses in the IBD and MDD transcriptomes separately. In the IBD cohort (135 IBD and 55 non-IBD samples), a total of 928 DEGS were identified, including 695 up-regulated genes and 233 down-regulated genes (Figure 1A). In the MDD cohort, 300 DEGs were obtained between the MDD group and the control group, including 117 up-regulated genes and 183 down-regulated genes (Figure 1B). Venn analysis further revealed 25 overlapping DEGs differentially expressed in IBD and MDD, which were identified as candidate genes involved in the common pathogenic process (Figure 1C).

### 3.2. Multi-Dimensional Functional Enrichment Analysis of 25 Shared DEGs

**Figure 2:**
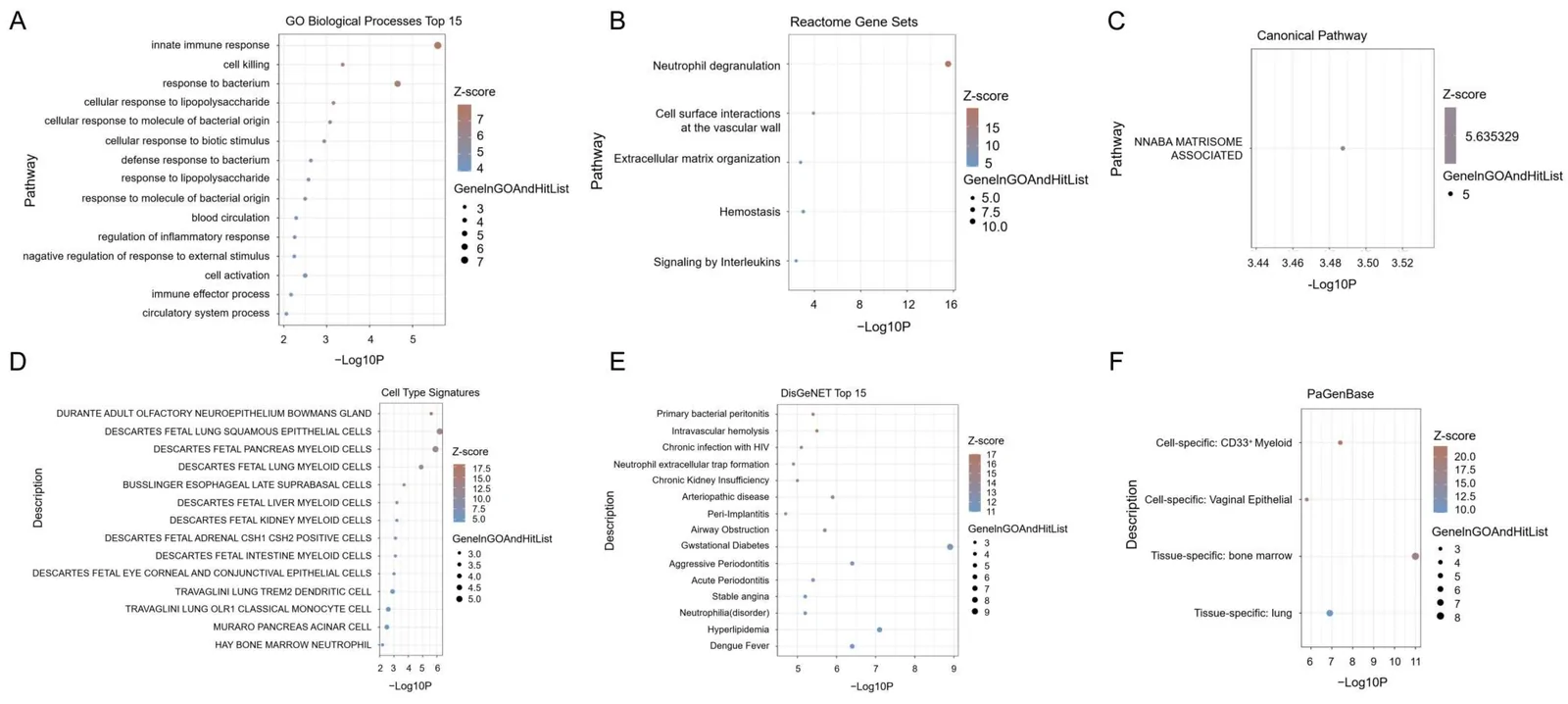
Bubble enrichment plots of six independent databases based on 25 overlapping DEGs between IBD and MDD. (**A**) Top 15 enriched GO Biological Process terms; (**B**) Reactome canonical pathway enrichment; (**C**) NABA matrisome-associated canonical pathway enrichment; (**D**) Cell type signature enrichment; (**E**) DisGeNET disease enrichment; (**F**) PaGenBase tissue/cell-specific enrichment. *X*-axis represents −log10 (*p*-value) for enrichment significance, dot color corresponds to Z-score (overall expression change trend of enriched genes), dot size indicates the number of hit genes enriched in each pathway.

To systematically explore the biological functions of these 25 shared genes, we performed six enrichment analyses. GO biological process enrichment showed that the shared genes were mainly enriched in innate immune response, inflammatory cell activation, and mucosal immune regulation (Figure 2A). Reactome pathway analysis highlights neutrophil degranulation, interleukin signaling, and extracellular matrix organization as core inflammatory pathways that are strongly associated with intestinal mucosal injury and neuroinflammation (Figure 2B). Enrichment of classical pathways further confirmed the marked activation of stroma-body remodeling pathways involved in tissue repair and inflammatory fibrosis (Figure 2C). Cell-signature enrichment indicated that these genes were strongly associated with immune cell infiltration and epithelial cell dysfunction (Figure 2D). In addition, DisGeNET disease enrichment verified their strong association with inflammatory bowel disease and neuropsychiatric disease (Figure 2E), while PaGenBase enrichment indicated their specific expression signature in gut and neural tissues (Figure 2F). Taken together, these results indicate that the common DEGs are mainly involved in immune inflammation and tissue homeostasis disruption, and these DEGs may be a key molecular bridge connecting intestinal inflammation and MDD-related neuroimmune disorders.

### 3.3. Multi-Machine Learning to Screen and Identify Common Hub Genes Shared by IBD and MDD

**Figure 3:**
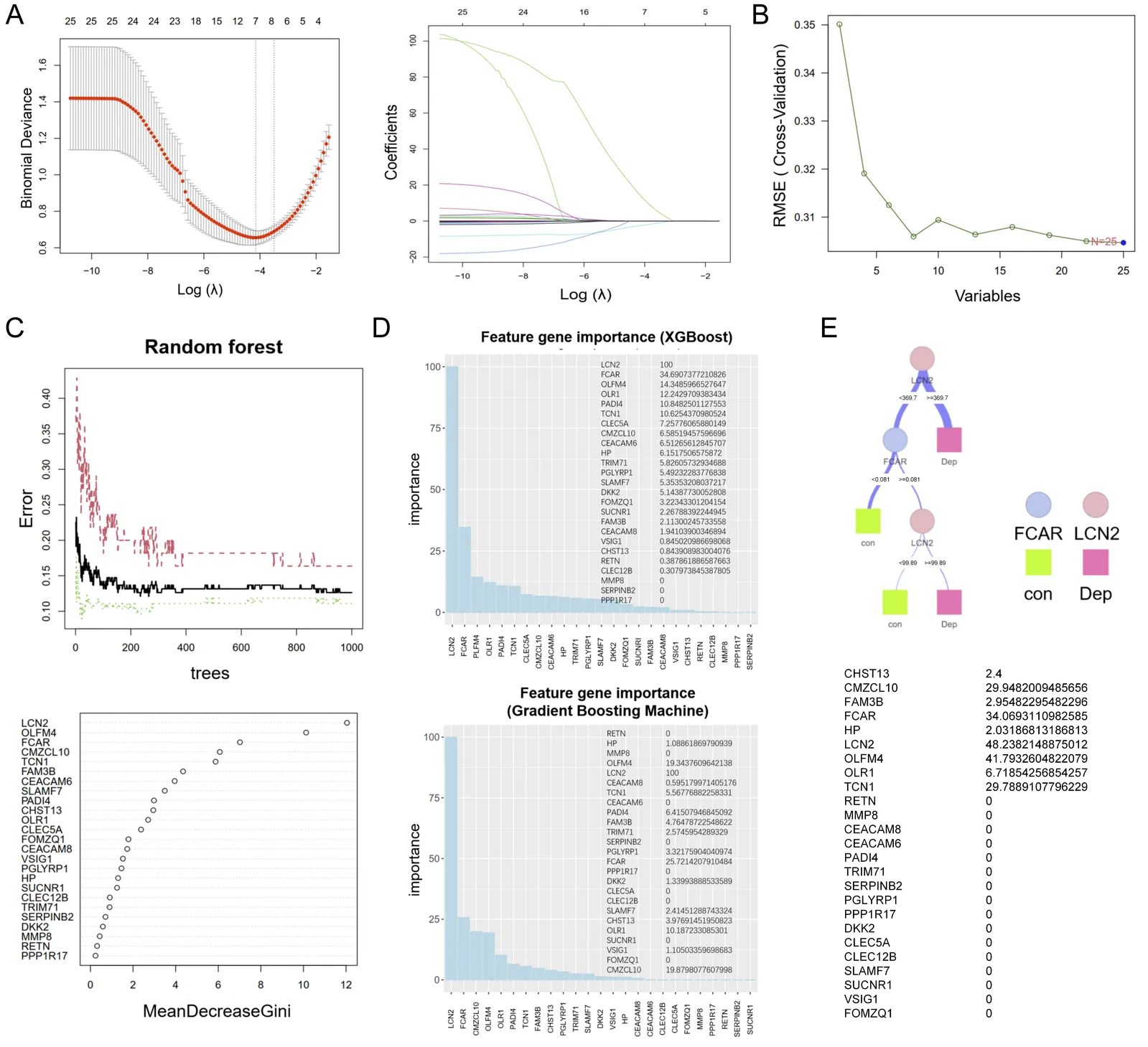
Multi-algorithm machine learning feature selection for IBD predictive biomarkers from 25 shared DEGs. (**A**) Left: LASSO binomial deviance cross-validation curve with λ.1se cutoff; Right: LASSO coefficient changing profile across varying log(λ). (**B**) Cross-validation RMSE curve for stepwise variable filtering. (**C**) Upper: Random forest error convergence curve with increasing tree numbers; Lower: Gene importance ranked by MeanDecreaseGini index. (**D**) Feature importance bar charts calculated by XGBoost (upper) and GBM (lower) algorithm respectively. (**E**) Simplified decision tree classification model of IBD based on screened core genes.

To screen stable and general diagnostic biomarkers for IBD and MDD from the 25 shared DEGs, we independently applied multiple machine learning algorithms including LASSO regression, random forest, XGBoost, GBM, and decision trees in the IBD and MDD cohorts. In the IBD cohort, seven candidate genes were selected by LASSO regression based on λ binomial bias cross-validation. The 1se criterion (Figure 3A), and the RMSE curve of stepwise regression confirmed the stability of feature selection (Figure 3B). The random forest model achieved stable convergence, ranking the importance of genes by the MeanDecreaseGini index (Figure 3C). The XGBoost and GBM algorithms further quantified the feature contribution of each candidate gene to IBD classification (Figure 3D) and constructed simplified decision trees to visualize the key predicted genes (Figure 3E).

**Figure 4:**
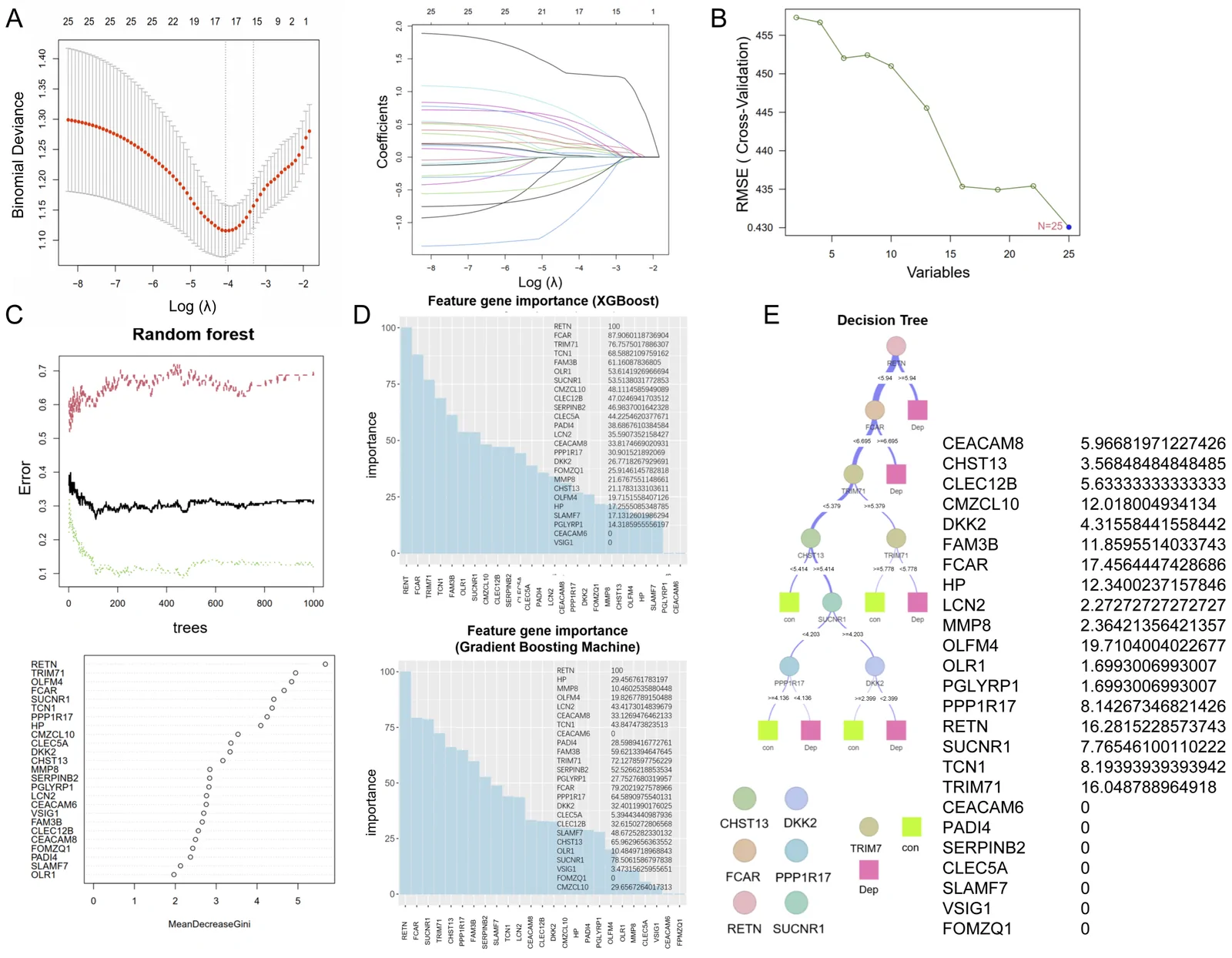
Multi-algorithm machine learning feature selection for MDD predictive biomarkers from identical 25 shared DEGs. (**A**) Left: LASSO cross-validation binomial deviance curve; Right: LASSO coefficient profile with changing regularization parameter λ. (**B**) Cross-validation RMSE curve for stepwise variable selection of MDD predictive features. (**C**) Upper: Random forest error convergence plot; Lower: Gene importance sorted by MeanDecreaseGini value. (**D**) Feature importance ranking plots derived from XGBoost (upper) and GBM (lower). (**E**) Decision tree predictive model constructed for MDD classification using filtered candidate genes.

For the MDD cohort, we used the same screening algorithm. LASSO regression similarly yielded seven candidate feature genes (Figure 4A), and RMSE curves verified the reliability of variable filtering (Figure 4B). Random forest convergence curves and gene-importance ranking identified core MDD-associated predictive genes (Figure 4C). The XGBoost and GBM models consistently quantified the predictive weight of each candidate gene for MDD identification (Figure 4D) and built a decision tree model for MDD classification (Figure 4E).

**Figure 5:**
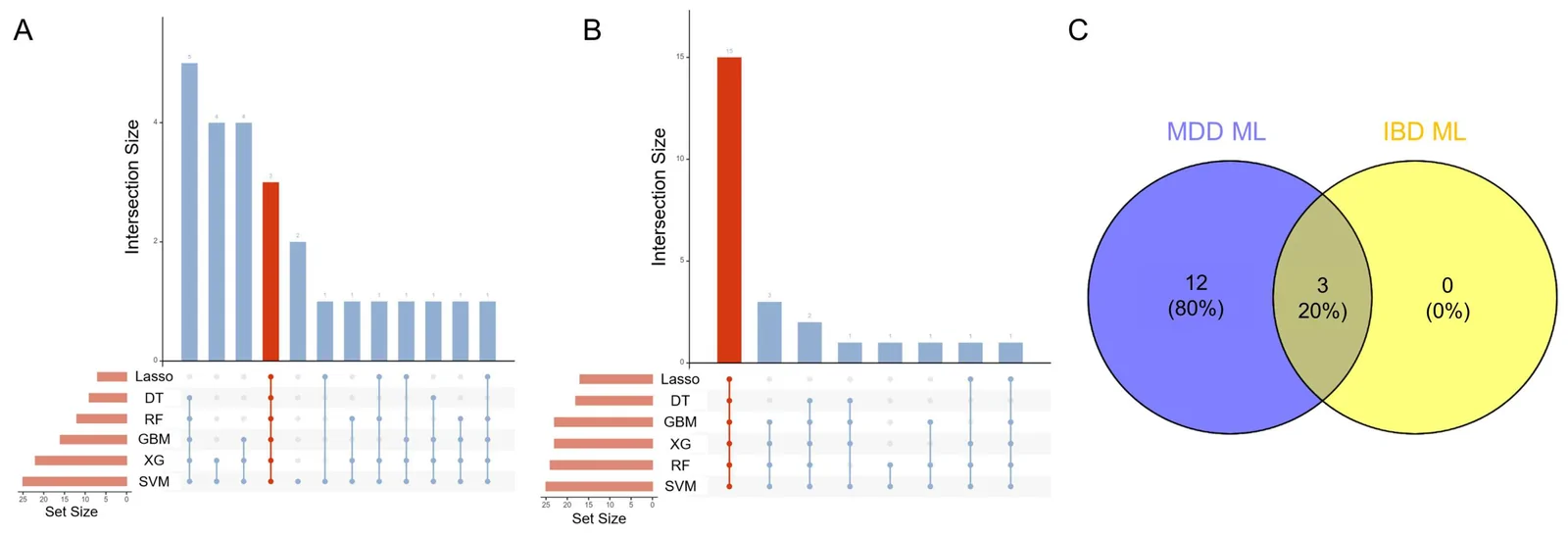
Upset plots and Venn diagram for overlapping machine learning-selected feature genes across IBD and MDD. (**A**) Upset plot showing overlapping genes among six machine learning models for IBD cohort; red vertical bar denotes consensus genes shared across multiple algorithms. (**B**) Upset plot displaying intersected genes among six ML models for MDD cohort. (**C**) Venn diagram of ML-screened signature genes between IBD and MDD; finally, 3 overlapping core hub genes (OLFM4, LCN2, FAM3B) were identified as shared biomarkers for both diseases.

In order to obtain stable hub genes that are generally applicable to these two diseases, we further intersected the machine learning-derived signature genes of the two cohorts. The flip plot shows overlapping and consistent genes between multiple algorithms in the IBD group (Figure 5A) and the MDD group (Figure 5B). Subsequent Venn intersection analysis ultimately identified three consensus core hub genes, OLFM4, LCN2, and FAM3B that were stably screened as key shared biomarkers for IBD and MDD (Figure 5C).

### 3.4. Marker Pathway Correlation Analysis of Three Core Hub Genes

**Figure 6:**
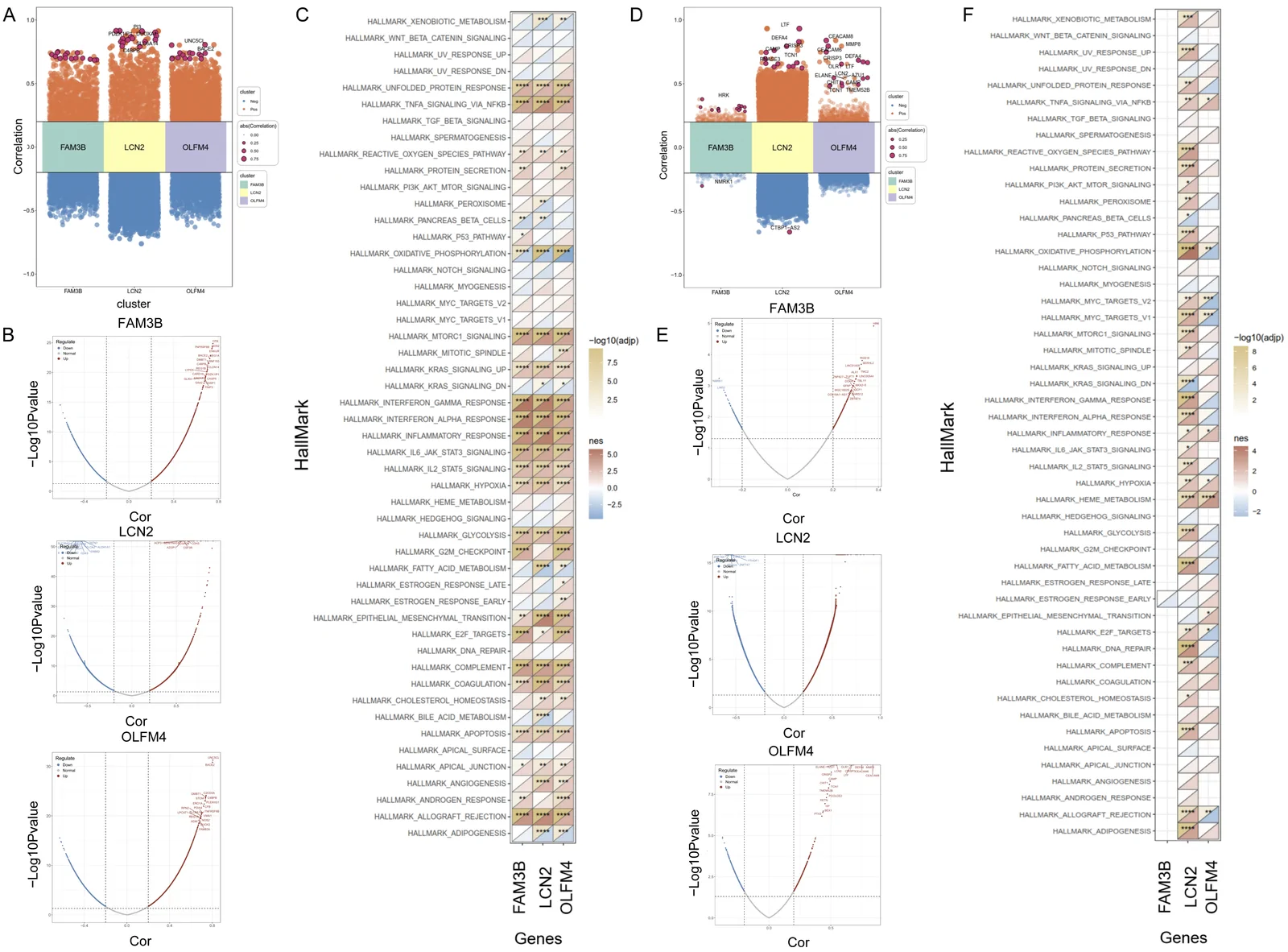
Hallmark gene set correlation analysis of OLFM4, LCN2 and FAM3B in IBD (**A**–**C**) and MDD (**D**–**F**). (**A**,**D**) Scatter plots displaying correlation between three hub genes and genome-wide transcripts; color gradient indicates positive (orange)/negative (blue) correlation. (**B**,**E**) Distribution curve of correlation coefficient and corresponding −log10 (*p*-value) for all correlated Hallmark pathways of each hub gene. (**C**,**F**) Heatmap-bubble plots of enriched Hallmark pathways, bubble color represents normalized enrichment score (NES), asterisk denotes statistical significance of enrichment. * *p* < 0.05, ** *p* < 0.01, *** *p* < 0.001, **** *p* < 0.0001.

To elucidate the potential regulatory mechanisms of OLFM4, LCN2, and FAM3B in IBD and MDD, we performed genome-wide gene association and marker pathway enrichment analyses in these two disease contexts. The gene association scatter plots showed that the three core genes were consistent with the global association pattern of the whole transcriptome genes in IBD (Figure 6A) and MDD (Figure 6D). The distribution of correlation coefficients and corresponding significance values further confirmed the stable association of hub genes with multiple functional pathways (Figure 6B,E). The signature pathway bubble heat map showed that these three genes were significantly associated with signaling pathways related to inflammatory response, immune activation, epithelial remodeling, and neuroinflammation in IBD and MDD (Figure 6C,F). OLFM4, LCN2 and FAM3B may mediate common immune-inflammatory diseases through the gut–brain axis, thereby contributing to the comorbidity of intestinal inflammation and depressive symptoms.

### 3.5. Correlation Between Core Genes and Immune Microenvironment Infiltration

**Figure 7:**
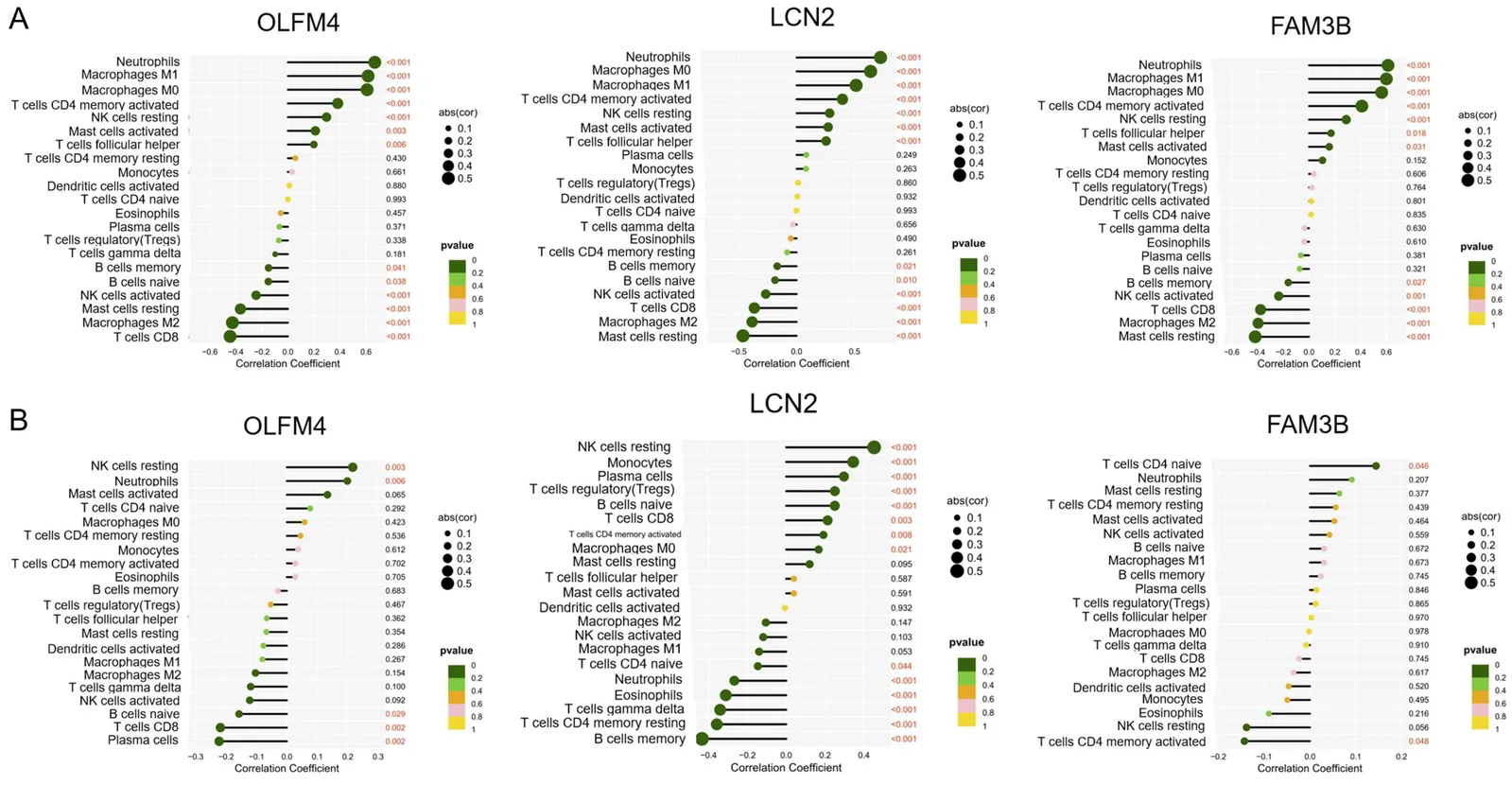
Correlation between core gene expression and immune cell infiltration in IBD and MDD. (**A**) Correlation coefficients between OLFM4, LCN2, FAM3B expression and 22 immune cell subsets in IBD cohort. (**B**) Correlation coefficients for the MDD cohort. The length of the horizontal line indicates the absolute value of the correlation coefficient, and the color of the dot indicates the significance level (*p*-value).

Correlations between the three hub genes and immune cell infiltration in IBD and MDD were analyzed using CIBERSORT-derived immune cell profiles (Figure 7). Taken together, these results show that these three hub genes are consistently associated with proinflammatory immune cell infiltration patterns, particularly neutrophils and M1-type macrophages, in both IBD and MDD, supporting their potential role that may contribute to gut–immune–brain axis related molecular alterations.

In the IBD cohort (Figure 7A), OLFM4, LCN2, and FAM3B expression was significantly and positively correlated with neutrophils and M1-type macrophages (all *p* < 0.001). In contrast, all three genes were negatively correlated with CD8^+^T cells and M2 macrophages (all *p* < 0.001). Other significant correlations were observed for other immune subsets, including a positive correlation with macrophage M0 and a negative correlation with quiescent mast cells.

In the MDD cohort (Figure 7B), LCN2 expression was strongly positively correlated with neutrophils (*p* < 0.001) and tended to be positively correlated with M1-type macrophages (*p* = 0.053). OLFM4 expression was also significantly positively correlated with neutrophils (*p* = 0.006). In contrast, in the MDD cohort, no significant association of FAM3B with neutrophils or M1 macrophages was observed.

Together, these results show that these three key genes are consistently associated with proinflammatory immune cell infiltration patterns, particularly neutrophils and M1 macrophages, in IBD and MDD, supporting their potential role in mediating gut–brain axis dysfunction.

### 3.6. Validation of the Diagnostic Performance of the Three-Gene Signature

**Figure 8:**
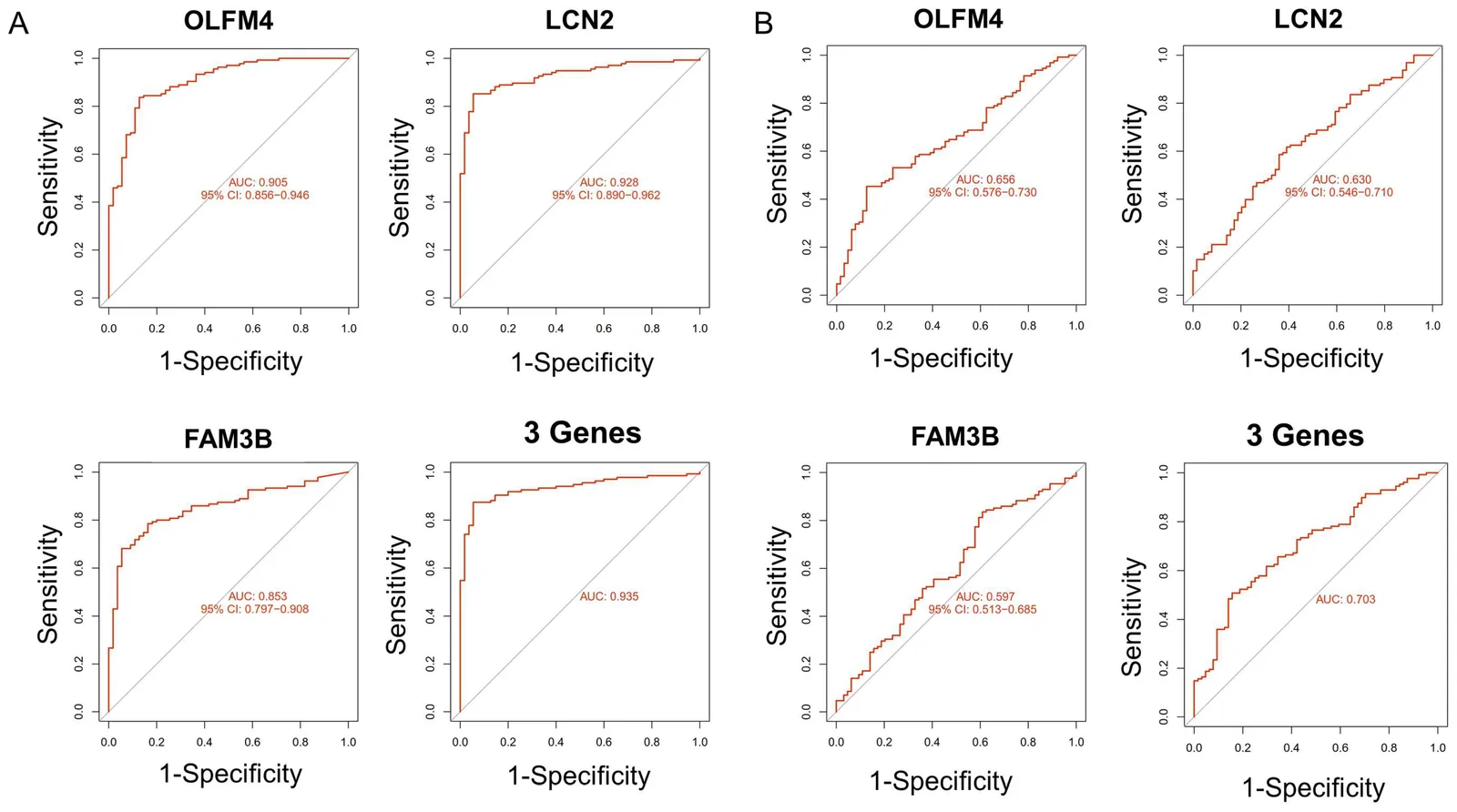
ROC curve analysis of the three-gene signature. (**A**) Diagnostic performance of OLFM4, LCN2, FAM3B, and their combined panel in distinguishing IBD patients from controls. (**B**) ROC curves for the same biomarkers in the MDD cohort. The AUC values with 95% confidence intervals are presented for each gene and the combined signature.

The diagnostic potential of individual core genes as well as the combined three-gene signature for IBD and MDD was assessed by ROC curve analysis (Figure 8). In the IBD cohort, OLFM4, LCN2 and FAM3B showed good independent diagnostic efficacy, and the three-gene joint model had the highest AUC value in distinguishing IBD and non-IBD samples (Figure 8A). In the MDD cohort, the joint signature also showed better diagnostic performance than the single gene, indicating its good discriminative power for MDD (Figure 8B). In conclusion, the OLFM4/LCN2/FAM3B signature shows preliminary discriminatory performance within these datasets for IBD and MDD. These genes represent candidate molecular signatures related to gut–immune–brain axis-associated inflammatory changes.

In this study, 25 shared DEGs based on IBD and MDD transcriptome profiles were systematically screened, and three consensus core genes (OLFM4, LCN2, FAM3B) were further identified by multi-algorithm machine learning filtering. Functional enrichment, marker pathway association, and immune microenvironment analyses revealed that these three genes mediate the inflammatory and immune dysregulation common to intestinal and nervous system diseases. ROC validation revealed preliminary discriminatory performance within each respective dataset. This study provides candidate shared molecular signatures and hypothetical mechanistic insights regarding molecular links between IBD and MDD, noting that our analysis does not include patients with clinical IBD-MDD comorbidity.

## 4. Discussion

Accumulating clinical evidence has confirmed a robust bidirectional comorbidity between IBD and MDD, where intestinal chronic inflammation exacerbates neuropsychiatric dysfunction, and depressive symptoms in turn accelerate IBD progression, forming a self-amplifying pathogenic cycle [16,17]. Current studies predominantly attribute this crosstalk to the dysregulated gut–immune–brain axis, which mediates systemic inflammatory spillover, neuroimmune activation and intestinal barrier dysfunction [18]. Nevertheless, high-quality prospective cohort studies and definitive shared molecular biomarkers for IBD-MDD comorbidity remain insufficient, severely hindering early intervention and precise clinical management of comorbid patients. In the present study, we systematically integrated pediatric IBD intestinal transcriptomic data and MDD peripheral blood data, and successfully screened three robust shared hub genes (OLFM4, LCN2, FAM3B) via differential analysis, multi-dimensional functional enrichment and multi-algorithm machine learning validation. Further pathway and immune infiltration analyses demonstrated that these three genes participate in IBD-MDD comorbidity by modulating inflammatory cascades and immune microenvironment homeostasis, providing novel objective biomarkers and mechanistic insights for gut–brain axis-mediated comorbid pathogenesis.

LCN2 is a member of the lipocalin superfamily. Increasing evidence shows that LCN2 is significantly up-regulated in various pathological microenvironments and actively participates in the pathogenesis and progression of a variety of diseases, including cancer, IBD, Alzheimer’s disease and MDD [19,20,21,22]. In IBD, the increased expression of LCN2 promotes mucosal inflammation and pyroptosis of intestinal epithelial cells by regulating the NF-κB/NLRP3/GSDMD signaling axis [22]. Another study has shown that hypoxia regulates the HIF-1α pathway through LCN2, which affects the polarization of M1 macrophages through glycolysis, thereby aggravating colitis [23]. A growing number of neuropsychiatric studies further confirm that LCN2 overexpression disrupts blood–brain barrier integrity, induces microglia hyperactivation, and triggers central neuroinflammation, which is the core pathological driver of depression onset and exacerbation [20,24]. Our immune correlation analysis confirmed that LCN2 positively correlated with proinflammatory M1 macrophages and neutrophils in both IBD and MDD cohorts, which is consistent with previous findings suggesting that LCN2 can simultaneously activate peripheral gut inflammation and central nervous immune imbalance, thereby promoting bidirectional disease progression.

OLFM4, another core secreted biomarker identified in this study, is closely involved in mucosal immune regulation and chronic inflammatory response [25,26]. In IBD, OLFM4 has multiple “mucin-like” properties (negatively charged polymerized glycoprotein, secreted in mucus, and bound to defensins) and is widely upregulated in the mucosa of inflammatory IBD, expanding to the surface epithelium and secreted into mucus. Induction may be mediated by bacteria through the Notch pathway and IL-22 [27]. In recent years, increasing evidence has shown that OLFM4 is abnormally expressed in MDD and is involved in its progression [28,29]. Our pathway analysis further verified that OLFM4 was closely associated with JAK-STAT and NF-κB inflammatory pathways, suggesting a pro-comorbid regulatory role in the peripheral gut and central nervous system.

FAM3B, also known as pancreatic derived factor (PANDER), is a cytokine-like protein that plays an important role in glucose metabolism and islet β cell function [30]. Recent transcriptomic and bioinformatics studies have begun to link FAM3B to inflammation and neuropsychiatric disorders, but direct mechanistic evidence for IBD and MDD remains limited [29]. In our analysis, FAM3B was differentially expressed in both IBD intestinal tissues and MDD-related samples, and co-expression network analysis suggested its involvement in pathways related to inflammation, cell adhesion, and extracellular matrix remodeling. In gastrointestinal inflammation, FAM3B can regulate epithelial cell survival and mucosal barrier function, and indirectly affect the severity of intestinal inflammation in IBD. For depression, a recent biomarker study proposed FAM3B as a potential diagnostic marker for MDD, implying that FAM3B may be associated with neuroinflammatory or metabolic pathways related to emotion regulation [31]. Notably, our ROC analysis showed that FAM3B alone and in combination with OLFM4 and LCN2 showed moderate diagnostic performance for both IBD and MDD, supporting its potential as a shared biomarker, although the precise molecular mechanism of FAM3B with gut–brain axis signaling requires further experimental validation.

Mechanistically, these three key genes may synergistically mediate IBD-MDD comorbidity through immune-inflammatory network. Functional enrichment showed that these genes were mainly enriched in TNF/NF-κB and JAK-STAT signaling pathways, which are classical pathways mediating gut–brain immune interactions. Intestinal inflammation driven by central gene dysregulation can induce systemic inflammation spilt and central neuroinflammation, while neuropsychiatric stress in turn aggravates intestinal immune dysfunction, forming a pathogenic cycle. Immune infiltration analysis further showed that these genes were associated with altered CD4^+^T cell, CD8^+^T cell and macrophage profiles, possibly bridging peripheral gut immune dysfunction and central nervous system immune dysfunction.

Our study has several limitations. First, all data were obtained from the public GEO database and the sample size was limited, which may have introduced potential false positive results and population heterogeneity. Second, our study analyzed two separate disease cohorts: intestinal biopsies from IBD only patients and whole blood samples from MDD-only patients. There were no samples from patients with true clinical IBD-MDD comorbidity. Further validation using samples from comorbid individuals is required, and cellular and animal experiments are needed to elucidate the causal mechanisms of these core genes in gut–brain axis-mediated comorbidity.

## 5. Conclusions

In conclusion, this study systematically identified three novel shared hub genes (OLFM4, LCN2, FAM3B) for IBD-MDD comorbidity by bioinformatics analysis. These core genes may participate in molecular crosstalk relevant to gut–brain axis dysfunction and exhibit preliminary discriminatory performance in individual disease datasets. This study provides new molecular biomarkers and mechanism reference for early diagnosis, mechanism exploration and targeted intervention of IBD combined with MDD.

## Data Availability

The datasets analyzed during the current study were obtained from the GEO public repository (GSE117993, https://www.ncbi.nlm.nih.gov/geo/, accessed on 30 March 2025; GSE98793, https://www.ncbi.nlm.nih.gov/geo/, accessed on 28 December 2024). No new datasets were generated in this study.
